# Biology of Pathogenic Microörganisms

**Published:** 1899-10

**Authors:** H. J. Washburn


### BIOLOGY OF PATHOGENIC MICROÖRGANISMS.

By Dr. H. J. Washburn.[1]

The subject under our consideration is not new, although most of the discoveries pertaining to it have been made since 1881. In the meantime a vast amount of work has been expended upon investigation into the life-history, habitat, and pathogenic properties of the various microbes.

It is certainly unnecessary for me, in this company, to undertake to establish the relations existing between certain forms of bacteria and certain diseases, or, in other words, to attempt to uphold the bacteria theory. There are plenty of people living to-day who scoff at the results of scientific research along this line, but I do not expect to find any of them here. We who have carefully raised cultures in the laboratory, and have watched the effect produced upon laboratory animals by inoculation from these cultures, and who have then brought our valuable assistant, the microscope, to bear upon the tissues of these animals at post-mortem, are left with but little room for doubting.

These minute forms are subject to the great rule of life which biology teaches us applies to all living things: They are born, they grow, they reproduce, and die. Like members of the human family, they affect those of their fellow-creatures with whom they come in contact, either to their improvement or their injury, and, taking into consideration the time required for the completion of their life-cycle, they certainly work out less destruction individually than some of the reasoning animals, who are subject to the same biological laws.

Microörganisms are of great interest to the veterinarian, and every discovery that relates to their life-history, or to the effects produced by them within the tissues of the higher orders of animals, is sure to be a help in understanding the course of the disease produced by the particular form under consideration. It must produce more satisfactory results from treatment given such patients, and leads us to more effective prophylactic measures.

After reading a work on bacteriology for a time, one is apt to think that every one of the numerous fleshy ills must be caused by some form of microbe, and we feel that the proper thing for the medical fraternity to do is to wage a war of extermination against the entire list of unicellular life. It is well not to be too hasty.

[1] Read before the meeting of the Missouri Valley Veterinary Association, June, 1899.

Further research will show us that only a small percentage of bacteria are harmful, the greater part being not only harmless, but of the greatest value to the advanced forms of life.

Leaving the useful ones, we will confine ourselves to those that produce discomfort, disease, or death when introduced under favorable conditions into the animal economy. These are the pathogenic microörganisms, and nearly all of them belong to the vegetable kingdom. They are usually grouped from a strictly morphological stand-point, but it would suit the requirements of the investigator into pathological conditions far better were they grouped according to their effects. It is well to know what is meant by bacilli, cocci, and spirilla, but it is *better* to know what to expect from the various members of these groups. As a rule, it may be stated that their deleterious effects are due to poisons produced by them, but there are a few exceptions. The *bacillus anthracis* often produces its most serious effects through the remarkable rapidity of its reproduction, and the consequent gorging and choking of the vascular system, rather than by direct poisoning. Pyogenic cocci may act in the same way.

Of the various diseases due to microörganisms, hog-cholera brings the greatest money-loss to the stockowners of the country; tuberculosis is the most feared by the well-informed, and rabies, assuming its cause to be a germ, is the most capable of filling the minds of the masses with a vague dread and terror.

Hog-cholera may serve us as a type of the severest bacterial disease which affects our domestic animals. Here we observe the characteristic fever, inflammation of digestive and respiratory organs, hemorrhagic lesions, and affected secretory surfaces. Severe, indeed, must be the suffering of a "cholera" hog. One who has lived through an attack of typhoid fever cannot for a long time afterward notice a hog with well-developed cholera without a returning realization of the suffering then endured. The poor hog plainly gives evidence of suffering from the same headache, the same dull, aching, watery, sticky eyes, the same intense fever, dysentery, painful micturition, with their accompanying anorexia, and disinclination to move. And why should he not? The bacillus of hog-cholera and the bacillus typhi abdominalis are very like in every way. They grow readily in the laboratory upon the common culture media, making a most luxuriant growth upon potato. They may be propagated with equal facility in milk, and it matters not whether this milk is in the incubator of a laboratory or in a crock on the pantry-shelf.

The colonies present macroscopically a dense centre with attenuated borders. There is nothing very characteristic or interesting in this, but just examine some well-prepared section from this colony under a good microscope, and how very quickly the whole scene changes. Of all the crazy, scurrying crowds, the one now brought to view takes the cake. They are described in the text-books as actively motile, and they thoroughly warrant such description. Each one hustles across the field in tumultuous haste, as though he believed a " bad black man " was right after him. No wonder that these organisms permeate the entire body of their victim, or that death so often steps in and ends their journeying.

Hog-cholera is one of the true septicæmic diseases. The organisms enter the general circulation and are conveyed to all vascular parts of the body. This advantage once gained, they multiply rapidly and cling to the vessel-walls wherever they are able to do so. They often gather in such numbers as to form plugs in the blood capillaries which result in hemorrhages. They lodge in the lymph-glands, in the capillary plexuses of the lungs, along the intestinal walls, and wherever lodged they produce their toxic secretions.

This organism offers extreme resistance to the effects of cold. Freezing does not seem to destroy it. Hence we must conclude that it is unsafe to depend on freezing wintry weather to stop an outbreak of cholera.

The theory has been advanced by Gaffky that these typhoid infections " must always occur through the mucous membranes of the intestines ; even when the poison seems to have been inhaled." He thinks it is caught on the mucous membranes of the pharynx, swallowed, carried through the stomach, and thus brought into contact with the intestine. From this point it enters the circulation and invades the lungs and other organs.

Leaving these organisms, let us briefly observe the action of the *tubercle bacillus*. This seems very reluctant to grow where it can be scrutinized at will by students. It is evidently too modest and retiring. It requires a specially prepared medium of blood-serum or glycerin-agar, and will not grow satisfactorily on the common bouillon, agar-agar, or gelatin. It prefers blood-serum, probably because this contains some element favorable to its growth, which the others lack.

Until very recently it has not been discovered leading a saprophytic existence, but the claim is now made by a scientific searcher living in southern France that he has discovered this bacillus living on some of the coarse, rank swamp-grasses growing near his home. We will, however, await with interest further developments of this discovery.

Its growth in the laboratory, even upon its preferred medium and under the most favorable conditions known, is so very slow that unless the culture is strictly pure the other organisms at once outgrow and destroy the specimens sought. For this reason several careful transplantings are advisable. This manner of growth is still characteristic of them after they have obtained a lodgment in one of the higher animals. The progress of the disease is insidious, slow, and evasive, and much time is required for a colony of them to become sufficiently scattered about as separate spots of death to cause their presence to be indicated by their host. Their action upon the tissues is purely toxic.

It has been suggested that some scrofulous conditions in the human subject may be due to a mixed infection, cocci having been inoculated with the tubercle bacilli. I would offer as a suggestion to those who may be in a position to experiment that possibly a method of curing recently established cases of tuberculosis might be found by making use of the troublous experiences of the laboratory worker, and allowing some rapidly growing and at the same time less destructive organism to overgrow and destroy the bacilli within the living body. The fact that the introduction of certain forms of bacteria into a system previously infected with other forms will kill one or both is well proven. Green, in his *Pathology*, says: "Recent experiments have shown that two microbes growing in the body may successfully oppose each other. Thus, if erysipelas cocci be injected under the skin and into the blood, and if a large dose of anthrax bacilli be introduced twenty-four hours afterward, so that a large number of the cocci are present at the time of infection, the anthrax bacilli will all die out in seventeen to twenty-four hours without causing even local œdema." The bacillus pyocyaneus is also antagonistic to the bacillus anthracis. An animal may be inoculated with a virulent anthrax-culture, and, if this be soon followed by an inoculation from a culture of bacillus pyocyaneus, the life of the anthrax bacillus is quickly destroyed.

So far as I have been able to learn, the cause of rabies has not been positively identified. Some bacteriologists in their efforts to get ahead of their fellows have declared positively that their research has been rewarded and that they have succeeded in locating the long-desired germ. But the amusing part of it is that no two of them agree on a description. One insists that it is a long, rod-like bacillus, another that it is short and blunt, but still a bacillus, while others have described it as a diplococcus. It has been variously proven to be motile and non-motile, aërobic and anaërobic, existing in the blood and not so existing. But as rabies is probably due to a germ, I will mention it here. The virulence, whatever it may be, is transmitted by the saliva, tears, milk, nervous tissues, etc., of the rabid patient, but never by the blood.

As I said earlier, rabies, more than any other of the diseases of the domesticated animals, fills the popular mind with dread and terror. What will be more anxiously or rapidly passed from mouth to mouth throughout a community than the reports incident to a "mad-dog scare?" This is one disease that it is unnecessary for any civilized land to harbor. A strict control of all dogs in the country for a season would readily exterminate rabies. This subject is being agitated somewhat in some of the Eastern sections of the United States. Federal control of all dogs for a short time, and the relentless destruction of all vagrant dogs, are suggested as a feasible way of ridding the greater part of the country of rabies for all time. Of course, there would remain danger from wolves, foxes, etc., in certain sections, but outbreaks from these sources need not spread far. I am wandering from my theme. This is not bacteriology.

The *tetanus bacillus* is another to claim our notice. It grows normally in rich garden-soil, and may be said to invade the animal system only by accident. For a long time all attempts to grow it in the laboratory were futile. Countless attempts were made, all sorts of media were tried, and various temperatures, but the results were unsatisfactory. At last a culture was placed in a hydrogen atmosphere and then it was found that the tetanus bacillus would thrive upon the common media and at a variety of temperatures. It was anaërobic. In raising cultures of this bacillus the culture-medium should be given a greater degree of alkalinity than is supplied to other varieties. The reaction may be quite decidedly alkaline. The growth of the colony is somewhat slow. They take the form of a dense, round centre with many delicate radii. A small amount of gas is produced as they grow, and a most intense poison as well. The presence of the bacillus is not necessary for the production of tetanus in animals. All that is required is the poisonous product that they furnish. Injection of animals with such product from a laboratory culture produces death from tetanus as quickly as though the living organisms themselves were introduced, and the symptoms and course of the disease are the same in either case.

From the laboratory growth of this bacillus we may learn at least two things. All wounds where the infection with the tetanus bacillus is suspected should be opened with a bold incision so as to admit air freely. Secondly, this should be done quickly, as the tetanic spasms are dependent upon what seems to be a fermentative action rather than upon the production of large numbers of microbes within the system.

In marked contrast to this organism is the growth of the *streptococcus pyogenes*. In the laboratory this microbe grows readily at first. It is easily started, and the colony soon presents a thriving appearance. But it soon seems to be receiving a check to its growth and to be losing its vitality. The cause is this : As it grows this colony excretes an acid which liquifies the adjoining bouillon and is rapidly fatal to the growing parasites. This results in the laboratory in inevitable death to the organisms—a sort of unavoidable suicide. They kill themselves, and cannot help it. Introduced into our patients the case is different. Here the blood-current supplies fresh food for them and removes the objectionable acid. This coccus is the great secondary invader. It follows surgical operations, and is the cause of spreading phlegmons, erysipelas, septicæmia, pericarditis, peritonitis, etc. This and the staphylococci are excellent organisms for the beginner in laboratory culture to attempt to grow. They start to grow quickly, are not over-particular as to the quality of the culture-media supplied them, will bear transplanting well, and are not dangerous to have on clothing or utensils in case the operator clumsily breaks his glassware while cultivating or examining his growing colonies.

They are much safer than the *bacillus anthracis*, which will next receive our attention. This bacillus is the best known of all bacteria. It was the first to be positively identified as a specific cause of disease. It has been the subject of continuous experimentation. It makes a ready growth upon any of the common media. The rods extend longitudinally as the colony of a thrifty culture grows, until they form long filaments which weave and twist themselves together so intimately that a large section from the culture may sometimes be lifted with the needle, and where the culture is forcibly divided the torn edges always present a very ragged appearance. The chief danger from the bacillus anthracis seems to arise when spores have been formed. Referring to this transformation, Ostertag says : "Of the greatest interest is the knowledge of the conditions most favorable to the sporulation of anthrax bacilli. Anthrax spores are only produced in the presence of a liberal supply of acids and under suitable conditions of temperature, the limits of favoring temperatures being 18° C. and 34° C., the most favorable being 30° C. But they will neither in the living animal body nor in the unmutilated carcass form spores. Once the spores are formed, however, they becomes a serious menace to all animal life that may come in contact with them. They will live for a long time in the earth or in stagnant water. Consequently low swampy places, and especially those that are subject to occasional overflow, are often in certain districts charged with anthrax spores for months and even years at a time. These spores, though inactive, are alive, and if again placed under favorable conditions, each spore will develop into a mature anthrax cell, and this cell in turn will rapidly multiply until myriads are produced.

Another of the motionless microbes, and one that is likewise dangerous for inexperienced parties to handle in any way, is the *bacillus mallei*, or the glanders bacillus. It grows better if the temperature of the incubator is kept rather low. As I have never grown any of the glanders bacillus, I will allow Abbott to furnish the following paragraph: "Its reactions to heat are very interesting. At 42° it will often grow for twenty days or more. It will not grow at 43° C., and is killed by exposure to this temperature for forty-eight hours. It is killed in five hours when exposed to 50° C., and in five minutes by 55° C. It grows both with and without oxygen; it is therefore facultative as regards its relation to this gas." Its action upon the tissues is quite similar to that of the tubercle bacillus, already mentioned, except that it acts far more rapidly, and the necrotic areas caused by it are characterized by a considerable formation of pus.

Before closing I wish to call your attention to a parasite noticed in a recent number of the *Breeders' Gazette*. The inquiry stated that the hogs in a certain section of Kentucky were at that writing affected with "a parasitic disease causing swelling of the lower jaw, eruptions on nose which enlarge until skin breaks, leaving the nose raw. After this the disease assumes the nature of a cancer. The nose rots away, the pig gets thin and weak, and dies." The reply was as follows: "This trouble is caused by a parasite resembling that of mange, and wherever it exists a poisonous matter seems to be produced." As any parasite answering the above description is a total stranger to me, I mention this here, hoping that any of you who have ever met him will hasten to introduce us.

This touches briefly a part of the pathogenic microörganisms. Many interesting forms are left unmentioned.

Many questions relative to the subject which we would like to have answered are still waiting for further investigation, and there is still room in this field for careful experimentation and for earnest study and thought.

*Discussion.*

Dr. Stewart: We certainly have had the pleasure of listening to an exceedingly interesting paper. To one who is not familiar with the problems presented they may seem somewhat difficult, but the author of them has made the understanding of them easier. There were points brought out in this paper which are somewhat new to me, or at least somewhat different from what I have been accustomed to think of them, and I would be very much pleased if the author would make some explanation before the discussion of this paper is closed.

If I remember correctly, the author stated that a variety of the micrococci behaved in the animal economy like anthrax bacilli; that they multiplied in the bloodstream, and adhere to the vessel walls, producing thrombi or infarcts with hemorrhage into the tissues. As I understand it, the ordinary micrococci do not multiply in the bloodstream, nor do they produce capillary hemorrhages.

The author classed hog-cholera as a variety of septicæmia, and this would seem a rather unusual classification. Many authors hold that one of the constant lesions found in septicæmic disease is enlargement of the spleen. I have seen many cases of what I term hog-cholera in which there were little or no splenic derangement, yet I will admit that in some cases of hog-cholera the spleen is enlarged. We have with us this evening one who is thoroughly posted on the subject, and I shall be pleased to have his ideas as to the classification of this disease. I refer to Dr. Peters, of Nebraska.

Dr. Peters: This is a pretty hot evening, but I must say that I have listened very intently to the doctor's paper, and enjoyed it very much. This is the first time that I can remember of a paper being read where I attended where they did not say something about swine-plague. The matter treated is very good. What the doctor has said about the hog-cholera bacillus meets to a great extent with my approval. I heard him quote my old friend, Gaffky, extensively, and I can assure you that with the exception of Dr. Smith and Shutz, of Germany, there are no men that have done as much thorough work. Now, the inspectors who are here this evening see hog-cholera galore, I think. They have their ideas.

The doctor gave you a very good idea of the bacillus of pneumonia. I am very sorry that he did not say something in regard to the swine-plague bacillus. May be it is very well that he did not, because I believe the time has come when we do not want to say too much about swine-plague. If I were to get to talking on hog-cholera, and go into details, I think some one would have to ring a bell, as I would not know when to stop.

There is probably no problem that confronts the sanitarian so forcibly as the question of hog-cholera. What can we do? I suppose you are all aware that Nebraska, for the last ten or twelve years, has made an attempt to do something to relieve the stockmen. Our State and the Bureau of Animal Industry have done a great deal of good within the last two years with antitoxin. There are many questions relating to this serum-work that need to be taken up and perfected, and I think that they will be in the near future. But the doctor has very clearly demonstrated in his paper how the hog-cholera bacillus grows, and how you can detect it, and, if any-one wants to make a culture, follow his directions.

Dr. Stewart has touched a very good point, and that is in regard to septicæmia and pyæmia. He has explained that, so I shall not do it. In regard to hog-cholera being a hemorrhagic septicæmia, I must say that I class it as such. I cannot put it in any other group.

Dr. Blackwell: Dr. Washburn mentioned something about an outbreak among hogs in Kentucky in which the jaw was affected. We have not heard anything on that question. I would like to hear from Dr. Peters.

Dr. Peters: That is a very interesting disease, and I think it is a variety of mange. We made some two hundred examinations of cases found in Nebraska, and we believe it is a parasite.

Dr. Stewart: Dr. Paul Fischer, of the Kansas State Agricultural College, investigated a peculiar contagious disease affecting the external genitals of cows. Dr. Conrad saw a number of cases of it. Dr. Fischer reported that he was able to get a culture of a vegetable organism, a trycophyton. He inoculated it in heifers, and succeeded in getting the characteristic pathological lesions. It occurred to me as a new rôle for the larger organisms.

Dr. Conrad: This disease was limited to a small area. I saw it in two bunches of cattle, and have not heard of any being reported from other points in Kansas.

Dr. Peters: There was one outbreak there where eight animals died. The peculiar part of it was that they were all black cattle, and the vagina was involved. The vagina was very highly inflamed, but I could not tell what the disease was. They would linger about three or four days and then die. I could not do anything with it.

Dr. Washburn: In reference to the matter of micrococci multiplying in the bloodstreams like the anthrax bacillus, I would say that this was demonstrated to us in our school as occurring when a virulent and thrifty young growth of the germ was inoculated into the guinea-pig.

